# A conditional denoising VAE-based framework for antimicrobial peptides generation with preserving desirable properties

**DOI:** 10.1093/bioinformatics/btaf069

**Published:** 2025-02-11

**Authors:** Weizhong Zhao, Kaijieyi Hou, Yiting Shen, Xiaohua Hu

**Affiliations:** Hubei Provincial Key Laboratory of Artificial Intelligence and Smart Learning, Central China Normal University, Wuhan, Hubei 430079, PR China; School of Computer, Central China Normal University, Wuhan, Hubei 430079, PR China; National Language Resources Monitoring & Research Center for Network Media, Central China Normal University, Wuhan, Hubei 430079, PR China; Hubei Provincial Key Laboratory of Artificial Intelligence and Smart Learning, Central China Normal University, Wuhan, Hubei 430079, PR China; Detroit Green Technology Institute, Hubei University of Technology, Wuhan, Hubei 430079, PR China; College of Computing & Informatics, Drexel University, Philadelphia, PA 19104, United States

## Abstract

**Motivation:**

The widespread use of antibiotics has led to the emergence of resistant pathogens. Antimicrobial peptides (AMPs) combat bacterial infections by disrupting the integrity of cell membranes, making it challenging for bacteria to develop resistance. Consequently, AMPs offer a promising solution to addressing antibiotic resistance. However, the limited availability of natural AMPs cannot meet the growing demand. While deep learning technologies have advanced AMP generation, conventional models often lack stability and may introduce unforeseen side effects.

**Results:**

This study presents a novel denoising VAE-based model guided by desirable physicochemical properties for AMP generation. The model integrates key features (e.g. molecular weight, isoelectric point, hydrophobicity, etc.), and employs position encoding along with a Transformer architecture to enhance generation accuracy. A customized loss function, combining reconstruction loss, KL divergence, and property preserving loss ensure effective model training. Additionally, the model incorporates a denoising mechanism, enabling it to learn from perturbed inputs, thus maintaining performance under limited training data. Experimental results demonstrate that the proposed model can generate AMPs with desirable functional properties, offering a viable approach for AMP design and analysis, which ultimately contributes to the fight against antibiotic resistance.

**Availability and implementation:**

The data and source codes are available both in GitHub (https://github.com/David-WZhao/PPGC-DVAE) and Zenodo (DOI 10.5281/zenodo.14730711).

## 1 Introduction

The accelerated growth and global spread of antimicrobial resistance have exacerbated the public health crisis (Magana *et al.* 2020). Moreover, the development of new antibiotics has significantly lagged behind market demand, and traditional drug development methods are increasingly inadequate to address the growing issue of antibiotic resistance. In this context, antimicrobial peptides (AMPs) have gradually gained attention as an effective alternative (Bucataru and Ciobanasu 2024).

AMPs are short, naturally occurring proteins with inhibitory activity against microorganisms, typically found in the lysosomes of macrophages and neutrophils (Rollins-Smith 2023). They can interact with the cell membranes of bacteria, fungi, viruses, or parasites, disrupting their membrane integrity and causing cell lysis and death (Li *et al.* 2022). Therefore, they hold the potential to address the growing health threat posed by multidrug-resistant microorganisms, attracting significant attention from the scientific community (Modiri *et al.* 2021). However, the natural sources of AMPs are limited, and they often face issues such as instability, short half-life, and severe hemolytic activity (Deo *et al.* 2022). Therefore, finding an efficient method to design new AMPs is in urgent demand.

Designing AMPs involves identifying amino acid sequences with antimicrobial properties from an extremely large sequence space. To address the challenge, various deep learning methods for AMP discovery have been proposed, including RNN-based (Muller *et al.* 2018, Shaon *et al.* 2024), LSTM-based (Mao *et al.* 2023), GAN-based (Tucs *et al.* 2020, Surana *et al.* 2023), and VAE-based (Dean *et al.* 2021, Pandi *et al.* 2023, Renaud and Mansbach 2023). These methods can effectively explore and generate diverse peptide sequences with potential antimicrobial activity, significantly expanding the sequence diversity of AMPs.

However, generating AMPs with desired properties remains a significant challenge. Although several conditional generation models (Szymczak *et al.* 2023, Hou *et al.* 2024, Wang *et al.* 2024) have been proposed for AMPs generation, the quality of generated samples still needs to be improved according to the biochemical perspective. Additionally, such models face another challenge, namely the issue of limited data, which leads to suboptimal performance in conditional generation of AMPs. Therefore, developing an efficient model for generating AMPs with desired physicochemical properties remains an urgent task that needs to be addressed.

In this paper, we propose a conditional denoising VAE-based model for AMP generation with desirable properties. This model comprehensively considers various physicochemical properties of AMPs, which determine AMPs’ biological activity and function, including antimicrobial efficacy, stability, target specificity, etc. More specifically, the physicochemical properties are embedded separately and are used to guide the training process of VAE’s encoder, which will transform the distribution of AMPs with required properties into a standard Gaussian distribution. In addition, the physicochemical properties are employed as well to train the decoder of VAE, by which AMPs with desirable properties can be generated by random samples from the standard Gaussian distribution. To address the challenges mentioned above, we incorporate Transformer and denoising techniques into the model. The Transformer model (Salem *et al.* 2022, Cao *et al.* 2023) has shown promising results in AMP synthesis. Specifically, its self-attention mechanism enables the model to capture comprehensive semantic relationships within amino acid sequences. Moreover, denoising techniques are applied during training to enhance the model’s generalization ability and prevent overfitting (Bengio *et al.* 2013). This approach introduces random perturbations, encouraging the latent space to learn to distinguish meaningful signals from noise. As a result, the representations in the latent space become more stable, addressing the issue of poor performance caused by data sparsity.

In summary, the contributions of the proposed model in generating AMPs are mainly summarized as follows.

- A novel conditional denoising VAE model is proposed for AMP generation, in which desirable physicochemical properties are utilized to effectively guide the training process of the encoder and decoder in VAE.
- With the incorporation of denoising techniques, the model can better handle noisy data during AMP generation, enhancing its robustness. This allows the model to generate high-quality AMPs with desired physicochemical properties, even when the data is limited.
- An innovative loss function is designed, which combines reconstruction loss, KL divergence, and property preserving (PP) loss, ensuring that the generated sequences retain the required physicochemical properties.
- Comprehensive experimental results demonstrate the model’s effectiveness in generating AMPs with desirable functional characteristics, providing a valuable tool for AMP design and analysis.

## 2 Materials and methods

**Figure 1.**
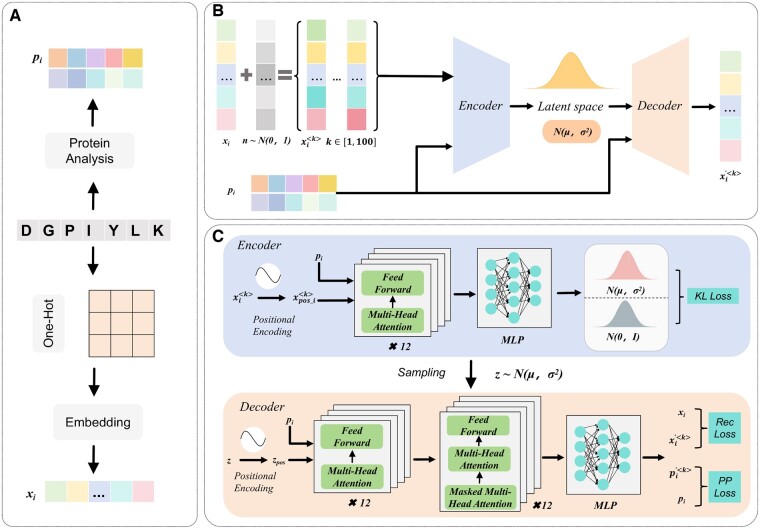
Workflow of the proposed framework. (A) Data processing. For each AMP sample *i*, we use the Protein Analysis tool to obtain 10 physicochemical properties of the AMPs and normalize them to obtain the property vector $p_{i}$; The sequence information of sample *i* is one-hot encoded and then passed through an Embedding layer to obtain the embedding vector$x_{i}$. (B) Basic framework. This is a conditional denoising VAE model. The input $x_{i}$ is added with Gaussian noise from a normal distribution to obtain the corresponding noised samples $x_{i}^{<k>}k\in [1,100]$. Both $x_{i}^{<k>}$ and $p_{i}$ are then passed through the encoder and decoder parts of the model, resulting in a reconstructed $x_{i}^{{}^{\prime }<k>}$, achieving the denoising effect. (C) Model training and inference. During inference, *z* is drawn from a standard normal distribution, while during training, it is sampled from the learned distribution N(μ, $\sigma^{2}$). MLP denotes a multi-layer perceptron. “KL Loss” measures the divergence between two probability distributions; “PP Loss” calculates the difference between the properties of the input and the reconstructed sequences; “Rec. Loss” represents the difference between the reconstructed sequence and the input sequence.

In this section, we provide a detailed description of the proposed AMP generation model, which is shown in Fig. 1. Each module of the proposed model will be described in the following subsections.

### 2.1 Data processing

This section aims to prepare the input for the conditional denoising VAE model, including high-dimensional embedding of sequence information and the extraction and normalization of AMPs’ features.

To better embed sequence information, we first apply one-hot encoding to the sequence data, representing each element as a sparse vector. However, directly using these sparse vectors can lead to low computational efficiency. Therefore, we introduce a pretrained embedding layer, which transforms the encoded data into dense representations. This allows the model to capture the latent relationships between sequence elements, providing more meaningful semantics.

For feature extraction, we utilize the Biopython (https://biopython.org) library to analyze the amino acid sequence and obtain relevant properties, including molecular weight, isoelectric point (pI), gravy, aromaticity, instability index, disulfide bonds, molecular volume, and secondary structure fraction (α-helix, β-sheet, and random coil). Note that the 10 selected properties are important for the biochemical function of peptides. Due to the space limit, the reason why selecting these 10 properties is described in detail in the Supplementary Material S1. To ensure dimensional consistency among the different features and avoid the impact of large numerical differences on model training, we normalize all extracted feature data. Specifically, we apply Min-Max normalization to scale the data to a unified range.

### 2.2 Conditional denoising VAE

The input of the model consists of sequence information and feature information from the data processing part. The main structure is composed of an encoder and a decoder. More specifically, the encoder maps the distribution of AMP space (i.e. the input data) to standard normal distribution, while the decoder transforms each sample generated from a standard normal distribution into the AMP sequence with desirable physicochemical properties (i.e. the input feature information). It is worth noting that the input feature information plays the role of guiding the training process of the VAE, by which the decoder can map the standard normal distribution to a conditional distribution of AMPs with desirable physicochemical properties.

#### 2.2.1 Adding noise

Due to the limited quantity of available data, the original VAE demonstrates suboptimal generative capabilities and struggles to effectively capture the underlying features present in the input data. This limitation adversely affects the model’s generalization ability, ultimately impacting its performance in real-world applications. To mitigate this issue, we have implemented a denoising process in training VAE. This method aims to enhance the model’s robustness and learning capacity by introducing noise to the embedding vectors during the training phase, thereby improving its ability to adapt to diverse data scenarios. Formally, given each sample $x_{i}$, the procedure of adding noise is defined as follows.

(1) $$x_{i}^{<k>}=\{\begin{array}{cc} x_{i}+N(0,\sigma^{2}) & \text{when}\;\text{training}\\ x_{i} & \text{when}\;\text{testing} \end{array}$$

where $x_{i}$ represents the input embedding vector, $x_{i}^{<k>}$ represents the kth noised embedding vector of $x_{i}$, and $N(0,\sigma^{2})$ denotes the noise drawn from a normal distribution with a mean of 0 and a deviation of 1. During the training phase, the output vector is influenced by noise, enhancing the model’s robustness; whereas in the testing phase, the output vector directly corresponds to the embedding vector. This approach ensures consistency in model performance across different stages while improving training effectiveness.

#### 2.2.2 Positional encoding

Since VAE and Transformer models are inherently order-agnostic, it is necessary to apply positional encoding for modeling AMP sequences. The self-attention mechanism in Transformer can capture the relationships between elements in the sequence, but it does not consider the order of elements. The positional encoding in the proposed model uses sine and cosine to generate encodings of different frequencies, defined formally as follows.

(2) $$PE(\mathrm{pos},2t)=\text{sin}\;\left(\frac{\mathrm{pos}}{10000^{\frac{2t}{d\_\text{model}}}}\right)$$

 

(3) $$PE(\mathrm{pos},2t+1)=\text{cos}\;\left(\frac{\mathrm{pos}}{10000^{\frac{2t}{d\_\text{model}}}}\right)$$

 

(4) $$x_{\mathrm{pos}\_i}^{<k>}=x_{i}^{<k>}+PE(\mathrm{pos})$$

where $x_{\mathrm{pos}\_i}^{<k>}$ represents the positionally encoded embedding vector for the sample $x_{i}^{<k>}$, *pos* denotes the position of the amino acid in the sequence, PE(pos) denotes the positional encoding generated using sinusoidal and cosinusoidal functions based on the position index *pos*, t represents the index of the dimension, and d_model represents the dimension of the embedding vectors. This ensures that the positional encoding vectors have different values at different positions and dimensions, capturing the positional information of the AMP sequences.

#### 2.2.3 Encoder

In the encoding part, the concatenated tensor is fed into a 12-layer Transformer encoder for feature extraction, in which it uses the self-attention mechanism to capture dependencies between elements in the sequence.

(5) $$x_{\mathrm{encoded}\_i}^{<k>}=\text{LayerNorm}(x_{\mathrm{pos}\_i}^{<k>}+\text{FFN}(\;\text{MultiHead}(x_{\mathrm{pos}\_i}^{<k>},p_{i})))$$

The sequence vector processed by the Transformer encoder is then pooled to obtain a fixed-length vector. The pooling operation is performed as follows.

(6) $${\bar{x}}_{\mathrm{encoded}\_i}^{<k>}=\frac{1}{L}\sum_{l=1}^{L}x_{\mathrm{encoded}\_i}^{<k>}$$

where ${\bar{x}}_{\mathrm{encoded}\_i}^{<k>}$ represents the pooled fixed-length vector, L represents the length of the sequence.

Finally, the fixed-length vector is passed through two fully connected layers with *ReLU* activation function to obtain the mean and variance of the transformed normal distribution (which denotes the latent embedding space of AMPs). The calculation formulas are defined as follows.

(7) $$\mu =W_{i}^{\mu }\cdot {\bar{x}}_{\mathrm{encoded}\_i}^{<k>}+b_{i}^{\mu }$$

 

(8) $$\sigma^{2}=W_{i}^{\mathrm{var}}\cdot {\bar{x}}_{\mathrm{encoded}\_i}^{<k>}+b_{i}^{\text{var}}$$

where μ represents the mean vector in the latent space, $W_{i}^{\mu }$ and $W_{i}^{\text{var}}$ are the weight matrices for the fully connected layers, $b_{i}^{\mu }$ and $b_{i}^{\text{var}}$ are the bias vectors for the fully connected layers, $\sigma^{2}$ denotes the variance vector in the latent space.

#### 2.2.4 Latent variable

In a VAE, the mean vector μ and the variance vector $\sigma^{2}$ produced by the encoder characterize the distribution in the latent space. In order to generate latent variable z following this distribution, sampling from this distribution is required. However, directly sampling from the parameterized Gaussian distribution poses difficulties, as it prevents gradient updates through backpropagation. To overcome this challenge, we employ the reparameterization trick (Kingma *et al.* 2015), which reformulates the sampling process into a differentiable operation.

First, during generation, the ϵ from the standard normal distribution is sampled from a Gaussian distribution with a mean of zero and a variance of ϵ∼N(0,I). Then, the mean vector μ and the standard deviation vector σ obtained by the encoder are used to reparameterize the latent variable z. More specifically, by using the reparameterization trick, the latent variable z is defined as follows.

(9) z=μ+σ·ϵ

#### 2.2.5 Decoder

In the decoding phase, we apply the comprehensive Transformer module, which includes both Transformer Encoder and Transformer Decoder. Specifically, the Transformer Encoder is used to integrate the conditional information, specifically latent variables and physicochemical properties. As for the Transformer Decoder, it is utilized to generate AMP sequences based on the encoded contextual representations. This architectural design enhances the model’s expressiveness and flexibility in handling complex mapping relationships.

First, the feature vector $p_{i}$ is concatenated with the latent variable z and passed through a fully connected layer to project it into a d_model space. The concatenated vector is first processed by a Transformer encoder, which consists of 12 layers, each containing a multi-head self-attention mechanism and a feed forward neural network. The specific formula is defined as follows.

(10) $$x_{\mathrm{encoded}\_i}^{<k>}=\text{LayerNorm}(z_{\mathrm{pos}\_i}^{<k>}+\text{FFN}(\;\text{MultiHead}(z_{\mathrm{pos}\_i}^{<k>},p_{i})))$$

where $x_{\mathrm{encoded}\_i}^{<k>}$ represents the value of the vector after passing through the Transformer encoder, and $z_{\mathrm{pos}\_i}^{<k>}$ denotes the latent variable value corresponding to the k-th noised result of the i-th sample after positional encoding.

Then the encoded sequence vector $x_{\mathrm{encoded}\_i}^{<k>}$ and $x_{i}$ are input into the Transformer Decoder.

(11) $$m_{i}^{<k>}=\text{MaskedMultiHead}(y_{i}^{<k>})$$

where $m_{i}^{<k>}$ represents the output of the masked-multi-head attention mechanism for the kth noised result of the ith sample, and $y_{i}^{<k>}$ represents the input at the current time step of the decoder.

(12) $$h_{i}^{<k>}=\text{MultiHead}(Q=m_{i}^{<k>},K=x_{\mathrm{encoded}\_i}^{<k>},V=x_{\mathrm{encoded}\_i}^{<k>})$$

where $h_{i}^{<k>}$ represents the output of the multi-head attention mechanism for the kth noised result of the ith sample. In this step, the Query (Q) is set to $m_{i}^{<k>}$, which is the result of the masked multi-head self-attention. The Key (K) and Value (V) are both taken from the Transformer encoder output $x_{\mathrm{encoded}\_i}^{<k>}$.

(13) $$x_{\mathrm{decoded}\_i}^{<k>}=\text{LayerNorm}(h_{i}^{<k>}+\text{FFN}(h_{i}^{<k>}))$$

For each position in the decoded $x_{\mathrm{decoded}\_i}^{<k>}$, which is to say, for each amino acid in the sequence, the position of the maximum value among the 21 values is determined. The formula is defined as follows.

(14) $$x_{i}^{\prime <k>}=\arg\max(x_{\mathrm{decoded}\_i}^{<k>})$$

where $x_{i}^{\prime <k>}$ represents the reconstructed data by taking the argument of the maximum value of $x_{\mathrm{decoded}\_i}^{<k>}$. By mapping the reconstructed indices to the corresponding amino acid letters, the reconstructed AMP amino acid sequence can be obtained.

### 2.3 Optimization

The proposed model is trained in an end-to-end manner, allowing it to automatically learn the mapping relationship between amino acid sequences and related properties without the need to manually design intermediate steps or features. In this study, the designed loss function consists of three components: KL loss, reconstruction loss, and PP loss. Firstly, the KL loss is defined as follows.

(15) $$\text{KL}\;\text{loss}=-0.5\left(1+\text{logvar}-\mu^{2}-\text{exp}(\text{logvar})\right)$$

Note that the KL loss plays a crucial role in VAE, which is used to measure the difference between the encoded latent distribution and the standard normal distribution, thereby guiding the model to transform the distribution of AMPs data into the standard normal distribution, which is simple to generate random samples.

Secondly, the reconstruction loss is defined by the following formula.

(16) $$\text{Rec}\;\text{loss}=\sum_{i=1}^{N}\sum_{k=1}^{100}\left(\text{CrossEntropy}(x_{i}^{\prime <k>},x_{i})\times \text{mask}\right)$$

Reconstruction loss is used to measure the difference between the model-generated $x_{i}^{\prime <k>}$ and the original input data $x_{i}$. By minimizing the reconstruction loss, the model can learn how to generate effective AMPs from the latent space. In practical implementation, “CrossEntropy” is used as a metric to measure the difference between the reconstructed data and the original data. By multiplying with the mask, only the loss of the actual data is calculated, ignoring the padding elements. This ensures that the model focuses more on the reconstruction of valid data during training, thereby improving the accuracy and quality of the reconstruction.

Finally, the PP loss is defined formally as follows.

(17) $$\text{PP}\;\text{loss}=\sum_{i=1}^{N}\sum_{k=1}^{100}\text{MSE}({p^{\prime }}_{i}{}^{<k>}p_{i})$$

PP loss is used to measure the difference between input properties and properties of reconstructed samples. By minimizing the PP loss, the model can learn a VAE which is able to generate samples (i.e. AMPs) with desirable properties (i.e. input properties). In practical implementation, mean squared error is used as the metric to calculate the difference between input properties and reconstructed properties.

By assigning different weights to the three loss functions, we obtain the final loss function, by which the model is guided to simultaneously optimize the structure of the latent space, the accuracy of reconstruction, and the properties of generated AMPs, thereby enhancing the overall performance of the model.

All parameters in our model are optimized using the Adam optimizer (Kingma and Ba 2014) and backpropagation in an end-to-end manner (Peng *et al.* 2021).

## 3 Experiments

In this section, we first provide a detailed description of the model training process, including data preparation, hyper-parameter settings, performance during training, and ablation study. We then evaluate our model by comparing it with existing models. In addition, we conduct comprehensive experiments to evaluate the proposed model, including the comparison between generated sequences and the training samples, the comparison between conditional generation and unconditional generation, and the comparison of generated sequences under different conditions. Finally, we design a case study to evaluate the ability of our model to discover new AMPs.

### 3.1 Training generative model

#### 3.1.1 Dataset

The AMP sequence data is collected from several related databases, including DRAMP (Shi *et al.* 2022), LAMP (Ye *et al.* 2020), and APD (Wang *et al.* 2016). A total of 21 350 entries are collected and divided into training, validation, and testing sets, with 14 925, 3198, and 3227 sequences, respectively. After removing AMPs with length >50, we finally obtained 12 489, 2650, 2700 sequences, respectively. All these sequences are analyzed using the Protein Analysis module to obtain 10 interested physicochemical properties which drastically affect the activity of AMPs. Finally, these sequences and the corresponding properties are combined for subsequent model training.

#### 3.1.2 Hyper-parameter setting

The implementation of the proposed model is based on PyTorch (https://pytorch.org). In our implementation, the encoder and decoder of Transformer both consist of 12 stacked layers, with 8 heads in the multi-head self-attention mechanism. The adding noise used in the denoising process follows a normal distribution with a mean of 0 and a deviation of 1. The embedding dimension d_model is set to 128, and the dropout rate is 0.1. During optimization, the learning rate is set to 0.0001, and the noise batch size is 100. These hyper-parameters are obtained through the hyper-parameter sensitivity analysis, the details of which are provided in the Supplementary Material S1. Before training, model parameters are initialized using Xavier (Glorot and Bengio 2010) initialization for linear layers, normal distribution initialization for embedding layers, and setting the weights of normalization layers to 1. This step ensures that the model is in a reasonable state at the start of training, which aids in stabilizing and accelerating the training process.

#### 3.1.3 Performance of training

To verify whether the model has learned the desirable latent representations of the data, we randomly select 8 dimensions from the 128-dimensional latent space each time and plot the resulting distribution accordingly. The learned distribution is then compared with the standard normal distribution, and the results are presented in Supplementary Fig. S2.

From the distribution shapes of these dimensions, we can see that the data generated by our model largely exhibits the characteristics of a normal distribution, with a mean of 0 and a variance close to 1. The variance values are marked in the top-right corner of each dimension, and overall, most variances are close to 1, indicating that the model has learned well and the difference between the latent space distribution and the standard normal distribution is really small. Due to the space limit, the detailed experimental analysis is presented in the Supplementary Material S1.

#### 3.1.4 Ablation study

To test the rationality of the proposed model, we conduct the ablation study in this section. More specifically, we evaluate the contribution of two components in our model, i.e. the denoising procedure and the PP loss. The results are presented in Supplementary Table S1.

Generally, the results demonstrate that both two components are helpful for improving the quality of generated AMPs. Due to the space limit, the detailed resultant analysis is provided in the Supplementary Material S1.

### 3.2 Overall comparison with baselines

To evaluate the proposed model, we compare it with six most representative models, including LSTM (Muller *et al.* 2018), AMP-GAN (Lin *et al.* 2021), PepGAN (Tucs *et al.* 2020), WAE (Renaud and Mansbach 2023), AMPEMO (Liu *et al.* 2023), and MoFormer (Wang *et al.* 2024). Due to the space limit, the basic introduction to selected baselines is presented in the Supplementary Material S1.

In this study, we evaluate AMP generation models according to hemolysis and toxicity, which are two critical factors in the development of AMP agents. High hemolysis and high toxicity of AMPs can lead to severe side effects in clinical settings. Therefore, effective or potential AMPs are required to have both hemolysis<0.5 and toxicity<0.5. In experiments, we sampled 1000 generated AMPs by each model, and compared the percentage of samples with the ideal factors. In addition, since our model requires the simultaneous input of 10 physicochemical properties, we randomly input values for these 10 properties each time (to simulate the unconditional generating process) to obtain the 1000 sequences. The comparison results are presented in Supplementary Table S2.

Generally, the results demonstrate that our model outperforms baselines in terms of hemolysis and toxicity. The detailed experimental analysis is presented in the Supplementary Material S1 due to the space limit.

### 3.3 Comparison between training samples and generated AMPs

In this section, we test if our model can learn the hidden semantic information contained in the training set. Specifically, we compare the distribution of sequence lengths and the distribution of amino acids in sequences between training samples and generated AMPs by our model. For training samples, we randomly select 1000 AMP sequences from the training set. As for generated AMPs, we use three settings of attribute values [obtained from Nisin (Gharsallaoui *et al.* 2016), Tachyplesin (Liu *et al.* 2018), and Temporin (Romero *et al.* 2020)] as conditional input in our model. For each setting of attribute values, 1000 AMPs are generated for comparison.

From Supplementary Fig. S3, we can find that training samples and generated AMPs share the similar distribution of sequence length. Specifically, the lengths of generated AMPs are primarily concentrated within the range of (0, 20], which is consistent with the typical length range of natural AMPs (Gagat *et al.* 2024). For the amino acid distribution in sequences, the comparison results are presented in Supplementary Fig. S4. The results in Supplementary Fig. S4 demonstrate that training samples and generated AMPs have similar distributions for some categories of amino acids while showing different patterns for some specific categories of amino acids (e.g., G, M, V, and W). By analyzing the biochemical characteristics of related amino acids, we draw the conclusion that our model can not only capture the hidden semantic information in the training set but also hold the potential to generate novel AMPs. Due to the space limit, the detailed resultant analysis is provided in the Supplementary Material S1.

### 3.4 Comparison of conditional and unconditional generation

To validate whether the generated AMPs by model exhibit required properties (i.e. the input desirable or expected properties), we compare the distribution of 10 physicochemical properties of AMPs obtained by conditional generation and unconditional generation, the results of which are shown in Supplementary Fig. S5. For each generation mode, we randomly sample 1000 AMPs to compute the distribution of 10 physicochemical properties. For conditional generation, the input condition is the physicochemical properties of one validated AMP *Tachyplesin*, for which the normalized feature vector is (0.34, 0.73, 0.45, 0.23, 0.16, 0.17, 0.34, 0.35, 0.05, 0.00). For unconditional generation, we run the proposed model with random conditions. Specifically, the input conditions are randomly sampled from the standard normal distribution to ensure that the generated AMPs have a certain degree of randomness to simulate unconditional generation. With this setup, we can thoroughly compare the differences between the random samples generated by the proposed model under specific condition and the random samples produced by the unconditional generation model.

Supplementary Figure S5 demonstrates that the distribution of physicochemical properties generated by the conditional generative model (blue curve) aligns more closely with the target value (indicated by the dashed blue vertical line), by comparing with the unconditional generative model (orange curve), which exhibits clear randomness and dispersion with the expected properties. Generally speaking, the result indicates that the proposed AMP generative model exhibits a clear advantage in preserving expected physicochemical properties and can be used as a feasible solution to generating AMPs with specific requirements. The detailed analysis of experiments is presented in the Supplementary Material S1 due to the limit of space.

### 3.5 Conditional generation results of different conditions

To further evaluate the generative performance of our model, we investigate the generated samples of our model under different input conditions. Specifically, we select three different settings of physicochemical properties (as shown in Supplementary Table S3), which are obtained from three validated AMPs (i.e., Nisin, Tachyplesin, and Temporin). Note that in this table, the 10 properties are in normalized values. To better demonstrate the difference between generated samples with different input properties, we employ t-SNE (Van der Maaten and Hinton 2008) to reduce the 10 properties into two dimensions, and plot the generated samples with different input properties by dots in different colors. To avoid mess for visualization, we select 50 AMPs for each condition, and the results are presented in Supplementary Fig. S6.

The data points in Supplementary Fig. S6 are clearly divided into three distinct clusters, which are represented in blue, green, and orange. These clusters indicate that AMPs with the same property setting are grouped together, with a noticeable separation between different categories. This observation demonstrates that our model can effectively produce desirable samples according to varying input conditions, further confirming that it can capture the underlying distribution of the AMPs through the effective training process.

### 3.6 Discovery of new AMPs

To further demonstrate the effectiveness of our model in practical applications, we customize the generation of AMPs with specific characteristics to replace those currently in use but have certain problems. In this study, we target Tachyplesin and generate AMPs similar to it with the aim of replacing it for potentially clinical use. Note that Tachyplesin is a widely used AMP. However, as its usage has expanded, issues such as toxicity, side effects, and resistance have gradually emerged, limiting its further promotion.

First, we utilize Protein Analysis to obtain the relevant physicochemical properties of Tachyplesin. Using these properties, we apply our model to generate a series of sequences, which are then screened for antimicrobial activity, toxicity, and hemolytic properties. The top 10 selected sequences are shown in Supplementary Table S4. After comparing the properties of these generated sequences with Tachyplesin, we can see that these sequences exhibit strong potential as substitutes. Specifically, most of the sequences exhibit strong similarities to Tachyplesin in key properties such as isoelectric point, instability index, and α-helical structure, with other characteristics also remaining largely consistent. Additionally, the biochemical properties of these 10 AMPs are analyzed, including AMP probability (Veltri *et al.* 2018), toxicity score (lower scores indicating lower toxicity) (Rathore *et al.* 2024), antibacterial activity, antiviral activity, and antifungal activity (Meher *et al.* 2017), and the results are shown in Supplementary Fig. S7. Moreover, the three-dimensional structures of six AMPs (as shown in Supplementary Fig. S8) are obtained by AlphaFold2 (Jumper *et al.* 2021). Generally, Seq1 and Seq5 exhibit relatively better performance across all metrics, making them the promising candidates to replace Tachyplesin. Due to the space limit, the detailed analysis is provided in the Supplementary Material S1.

According to the above discussions about finding potential alternatives to Tachyplesin among generated AMPs, we focus on generating potentially effective AMPs against antibiotic-resistant bacteria. Specifically, we conduct experiments targeting three resistant strains: Staphylococcus aureus, Escherichia coli, and Mycobacterium tuberculosis. We utilize Hdock (Yan *et al.* 2020) for molecular docking experiments to evaluate the interactions between predicted compounds and target proteins. The docking score, a key metric in this analysis, is calculated using the iterative scoring functions ITScorePP or ITScorePR. A more negative docking score indicates a higher likelihood of binding, with scores around -200 or lower being desirable. A confidence score above 0.7 suggests a high probability of binding between the two molecules; scores between 0.5 and 0.7 indicate possible binding, while scores below 0.5 suggest low likelihood of interaction.

**Figure 2.**
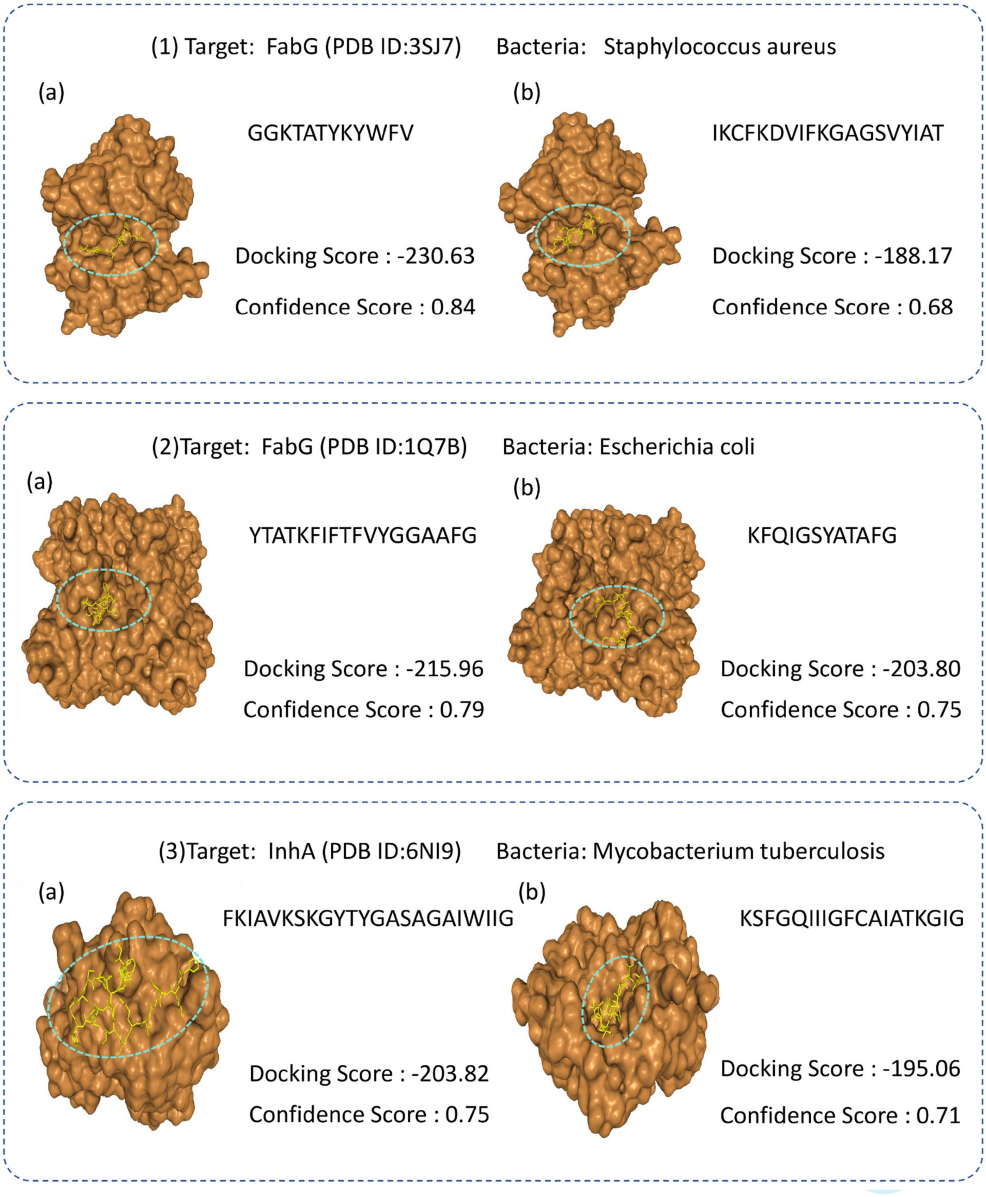
Results of molecular docking. (1) The docking results for the target protein FabG (PDB ID: 3SJ7) of *Staphylococcus aureus*; (2) the docking results for the target protein FabG (PDB ID: 1Q7B) of *Escherichia coli*; (3) the docking results for the target protein InhA (PDB ID: 6NI9) of *Mycobacterium tuberculosis*.

For the AMPs effective against *Staphylococcus aureus* and *Escherichia coli*, we reference Tachyplesin, a cationic β-hairpin AMP discovered approximately 30 years ago in the horseshoe crab. It is renowned for its potential antibacterial activity against multidrug-resistant bacteria and its cytotoxicity toward mammalian cells. Tachyplesin inhibits the enzyme activity of FabG by binding to it, thereby affecting bacterial survival. The results of our docking experiments with generated AMPs are presented in Fig. 2 (panels 1 and 2), where the docking scores and confidence scores meet the established criteria. Notably, the docking score for the experiment shown in panel 1(a) is −230.63, with a confidence score reaching 0.84. For AMPs targeting Mycobacterium tuberculosis, we reference Temporin L (Mineo and Halim 2024), a class of small linear AMPs extracted from the skin of the European red frog. Previous studies (Mineo and Halim 2024) have demonstrated its strong inhibitory effects against Mycobacterium tuberculosis. The docking results for the selected AMPs against this bacterium are shown in Fig. 2 (panel 3), where the docking scores and confidence scores also align with the screening criteria.

Although further investigations such as wet-lab experimental validation are required, the above observations indicate that the proposed model holds the potential to provide solutions to designing effective AMPs for addressing antibiotic-resistant pathogens.

## 4 Conclusions and future work

In this study, we have proposed a novel framework for generating AMPs based on VAE, Transformer architecture, and denoising techniques, aimed at generating peptide sequences with preserving specific physicochemical properties. Specifically, during training, we first add normal noise to high-dimensional vectors obtained from one-hot encoded sequences, effectively addressing the issue of limited training data. The sequence information, enhanced by positional encoding, is concatenated with feature information and processed through the Transformer encoder to obtain a representation in a transformed latent space. Hidden variables drawn from the latent space and the desirable properties are concatenated, and then passed through the Transformer-based decoder to obtain the reconstructed AMP sequences. A designed loss function is employed to optimize the whole model. During generation, the latent variables are sampled from a standard normal distribution, which are then concatenated with the expected properties to generate the peptide sequences via the Transformer-based decoder. We conduct comprehensive experiments to verify the effectiveness of the proposed model. Experimental results demonstrate that the model can generate AMPs with desirable physicochemical properties while maintaining comparable or better performance to other models in terms of key attributes such as toxicity and hemolytic activity, thus providing a potential solution to addressing the issue of antibiotic resistance.

However, there is still some room for improving in property preservation and antimicrobial activity of generated AMPs. In future, we plan to apply the pretrain plus fine-tuning procedure to optimize the generative model. Specifically, the generated model is first pretrained on more extensive peptides (not limiting AMPs) to learn more comprehensive semantics among sequences of amino acids. The pretrained model is then fine-tuned on high-quality data of AMPs to capture the specific semantics contained in biochemically active AMPs.

## Supplementary Material

btaf069_Supplementary_Data

## References

[btaf069-B1] Bengio Y , Yao L, Alain G et al Generalized denoising auto-encoders as generative models. Adv Neur Inf Process Syst 2013;26.

[btaf069-B2] Bucataru C , Ciobanasu C. Antimicrobial peptides: opportunities and challenges in overcoming resistance. Microbiol Res 2024;286:127822. page 38986182 10.1016/j.micres.2024.127822

[btaf069-B3] Cao Q , Ge C, Wang X et al Designing antimicrobial peptides using deep learning and molecular dynamic simulations. Brief Bioinform 2023;24:bbad058. 36857616 10.1093/bib/bbad058

[btaf069-B4] Dean SN , Alvarez JAE, Zabetakis D et al Pepvae: variational autoencoder framework for antimicrobial peptide generation and activity prediction. Front Microbiol 2021;12:725727. 34659152 10.3389/fmicb.2021.725727 PMC8515052

[btaf069-B5] Deo S , Turton KL, Kainth T et al Strategies for improving antimicrobial peptide production. Biotechnol Adv 2022;59:107968. 35489657 10.1016/j.biotechadv.2022.107968

[btaf069-B6] Gagat P , Ostrówka M, Duda-Madej A et al Enhancing antimicrobial peptide activity through modifications of charge, hydrophobicity, and structure. Int J Mol Sci 2024;25:10821. 39409150 10.3390/ijms251910821 PMC11476776

[btaf069-B7] Gharsallaoui A , Oulahal N, Joly C et al Nisin as a food preservative: part 1: physicochemical properties, antimicrobial activity, and main uses. Crit Rev Food Sci Nutr 2016;56:1262–74. 25675115 10.1080/10408398.2013.763765

[btaf069-B8] Glorot X , Bengio Y. 2010. Understanding the difficulty of training deep feedforward neural networks. In: *Proceedings of the Thirteenth International Conference on Artificial Intelligence and Statistics*, pp. 249–256. JMLR Workshop and Conference Proceedings.

[btaf069-B9] Hou K , Weizhong Z, He HT. 2024. Physicochemical property-guided conditional vae for antimicrobial peptides generation. In: *Proceedings of IEEE International Conference on Bioinformatics and Biomedicine*. IEEE.

[btaf069-B10] Jumper J , Evans R, Pritzel A et al Highly accurate protein structure prediction with alphafold. nature 2021;596:583–9. 34265844 10.1038/s41586-021-03819-2 PMC8371605

[btaf069-B11] Kingma DP , Ba J. Adam: a method for stochastic optimization. arXiv preprint arXiv:1412.6980, 2014, preprint: not peer reviewed. 10.48550/arXiv.1412.6980

[btaf069-B12] Kingma DP , Salimans T, Welling M. Variational dropout and the local reparameterization trick. Adv Neur Inf Process Syst 2015;28.

[btaf069-B13] Li X , Zuo S, Wang B et al Antimicrobial mechanisms and clinical application prospects of antimicrobial peptides. Molecules 2022;27:2675. 35566025 10.3390/molecules27092675 PMC9104849

[btaf069-B14] Lin T-T , Yang L-Y, Wang C-T et al Discovering novel antimicrobial peptides in generative adversarial network. BioRxiv 2021:2021-11, preprint: not peer reviewed. 10.1101/2021.11.22.469634

[btaf069-B15] Liu C , Qi J, Shan B et al Tachyplesin causes membrane instability that kills multidrug-resistant bacteria by inhibiting the 3-ketoacyl carrier protein reductase fabg. Front Microbiol 2018;9:825. 29765362 10.3389/fmicb.2018.00825 PMC5938390

[btaf069-B16] Liu Y , Zhang X, Liu Y et al Evolutionary multi-objective optimization in searching for various antimicrobial peptides [feature]. IEEE Comput Intell Mag 2023;18:31–45.

[btaf069-B17] Magana M , Pushpanathan M, Santos AL et al The value of antimicrobial peptides in the age of resistance. Lancet Infect Dis 2020;20:e216–e230. 32653070 10.1016/S1473-3099(20)30327-3

[btaf069-B18] Mao J , Guan S, Chen Y et al Application of a deep generative model produces novel and diverse functional peptides against microbial resistance. Comput Struct Biotechnol J 2023;21:463–71. 36618982 10.1016/j.csbj.2022.12.029 PMC9804011

[btaf069-B19] Meher PK , Sahu TK, Saini V et al Predicting antimicrobial peptides with improved accuracy by incorporating the compositional, physico-chemical and structural features into Chou’s general pseaac. Sci Rep 2017;7:42362. 28205576 10.1038/srep42362 PMC5304217

[btaf069-B20] Mineo M , Halim M. Synthesis and characterization of antimicrobial peptides targeting the DNA gyrase of *Mycobacterium tuberculosis*. 2024. https://digitalcommons.kennesaw.edu/undergradsymposiumksu/spring2024/spring2024/254/

[btaf069-B21] Modiri S , Kermanshahi RK, Soudi MR et al Growth optimization of lactobacillus acidophilus for production of antimicrobial peptide acidocin 4356: scale up from flask to lab-scale fermenter. Iranian Journal of Biotechnology 2021;19:e2686. 34825011 10.30498/ijb.2021.218725.2686 PMC8590721

[btaf069-B22] Muller AT , Hiss JA, Schneider G. Recurrent neural network model for constructive peptide design. J Chem Inf Model 2018;58:472–9. 29355319 10.1021/acs.jcim.7b00414

[btaf069-B23] Pandi A , Adam D, Zare A et al Cell-free biosynthesis combined with deep learning accelerates de novo-development of antimicrobial peptides. Nat Commun 2023;14:7197. 37938588 10.1038/s41467-023-42434-9 PMC10632401

[btaf069-B24] Peng J , Wang Y, Guan J et al An end-to-end heterogeneous graph representation learning-based framework for drug–target interaction prediction. Brief Bioinform 2021;22:bbaa430. 33517357 10.1093/bib/bbaa430

[btaf069-B25] Rathore AS , Choudhury S, Arora A et al Toxinpred 3.0: an improved method for predicting the toxicity of peptides. Comput Biol Med 2024;179:108926. 39038391 10.1016/j.compbiomed.2024.108926

[btaf069-B26] Renaud S , Mansbach RA. Latent spaces for antimicrobial peptide design. Digital Discov 2023;2:441–58.

[btaf069-B27] Rollins-Smith LA. The importance of antimicrobial peptides (amps) in amphibian skin defense. Dev Comp Immunol 2023;142:104657. 36754220 10.1016/j.dci.2023.104657

[btaf069-B28] Romero SM , Cardillo AB, Martínez Ceron MC et al Temporins: an approach of potential pharmaceutic candidates. Surg Infect (Larchmt) 2020;21:309–22. 31804896 10.1089/sur.2019.266

[btaf069-B29] Salem M , Keshavarzi Arshadi A, Yuan JS. Ampdeep: hemolytic activity prediction of antimicrobial peptides using transfer learning. BMC Bioinformatics 2022;23:389. 36163001 10.1186/s12859-022-04952-z PMC9511757

[btaf069-B30] Shaon MSH , Karim T, Sultan MF et al Amp-rnnpro: a two-stage approach for identification of antimicrobials using probabilistic features. Sci Rep 2024;14:12892. 38839785 10.1038/s41598-024-63461-6 PMC11153637

[btaf069-B31] Shi G , Kang X, Dong F et al Dramp 3.0: an enhanced comprehensive data repository of antimicrobial peptides. Nucleic Acids Res 2022;50:D488–D496. 34390348 10.1093/nar/gkab651 PMC8728287

[btaf069-B32] Surana S , Arora P, Singh D et al Pandoragan: generating antiviral peptides using generative adversarial network. SN Comput SCI 2023;4:607.

[btaf069-B33] Szymczak P , Możejko M, Grzegorzek T et al Discovering highly potent antimicrobial peptides with deep generative model hydramp. Nat Commun 2023;14:1453. 36922490 10.1038/s41467-023-36994-z PMC10017685

[btaf069-B34] Tucs A , Tran DP, Yumoto A et al Generating ampicillin-level antimicrobial peptides with activity-aware generative adversarial networks. ACS Omega 2020;5:22847–51. 32954133 10.1021/acsomega.0c02088 PMC7495458

[btaf069-B35] Van der Maaten L , Hinton G. Visualizing data using t-SNE. J Mach Learn Res 2008;9:2579–605.

[btaf069-B36] Veltri D , Kamath U, Shehu A. Deep learning improves antimicrobial peptide recognition. Bioinformatics 2018;34:2740–7. 29590297 10.1093/bioinformatics/bty179 PMC6084614

[btaf069-B37] Wang G , Li X, Wang Z. Apd3: the antimicrobial peptide database as a tool for research and education. Nucleic Acids Res 2016;44:D1087–D1093. 26602694 10.1093/nar/gkv1278 PMC4702905

[btaf069-B38] Wang L , Fu X, Yang J et al Moformer: multi-objective antimicrobial peptide generation based on conditional transformer joint multi-modal fusion descriptor. arXiv, arXiv:2406.02610, 2024, preprint: not peer reviewed.

[btaf069-B39] Yan Y , Tao H, He J et al The hdock server for integrated protein–protein docking. Nat Protoc 2020;15:1829–52. 32269383 10.1038/s41596-020-0312-x

[btaf069-B40] Ye G , Wu H, Huang J et al Lamp2: a major update of the database linking antimicrobial peptides. Database 2020;2020:baaa061. 32844169 10.1093/database/baaa061 PMC7447557

