# Supplementary material for: A conditional denoising VAE-based framework for antimicrobial peptides generation with preserving desirable properties
Source: Bioinformatics. 2025 Feb 11;41(2):btaf069. doi: 10.1093/bioinformatics/btaf069 (PMC11850229; doi:10.1093/bioinformatics/btaf069)
Supplement: btaf069_Supplementary_Data [file btaf069_supplementary_data.pdf]

Supplementary Material for "A Conditional Denoising VAE-based Framework for Antimicrobial Peptides Generation with Preserving Desirable Properties"

1 Detailed Reasons for Selecting the ten Properties of AMPs in Our Study

In this study, we select ten important properties of AMPs as the generative guidance, including Molecular Weight, Isoelectric Point (pI), Gravy, Aromaticity, Instability Index, Disulfide Bonds, Molecular Volume, and Secondary Structure Fraction (α-helix, β-sheet, and random coil). It is worth noting that the ten selected properties are all important for the biochemical function of peptides. Specifically, the molecular weight and molecular volume have been shown to play an important role in tuning the antimicrobial activity and selectivity, especially in regulating the specificity between Gram-negative and Gram-positive bacteria (Jiang *et al.*, 2021); the isoelectric point determines the peptides' solubility and interaction with cell membranes under varying pH environments, thereby affecting their antimicrobial activity (Hitchner *et al.*, 2019); aromaticity and disulfide bonds enhance the peptides' structural stability and resistance to degradation, ensuring their efficacy in complex biological environments (Chen *et al.*, 2017); the instability index aids in predicting the peptides' in vivo stability, guiding sequence optimization to extend their half-life (Yang *et al.*, 2024); meanwhile, the secondary structure score is directly associated with the peptides' functional mechanism, determining their mode of interaction with bacterial membranes (Rydberg *et al.*, 2012). By comprehensively modeling these properties, the model can design antimicrobial peptides that are more efficient, stable, and rationally optimized.

2 Supplementary Material for Hyper-parameter Sensitivity Analysis

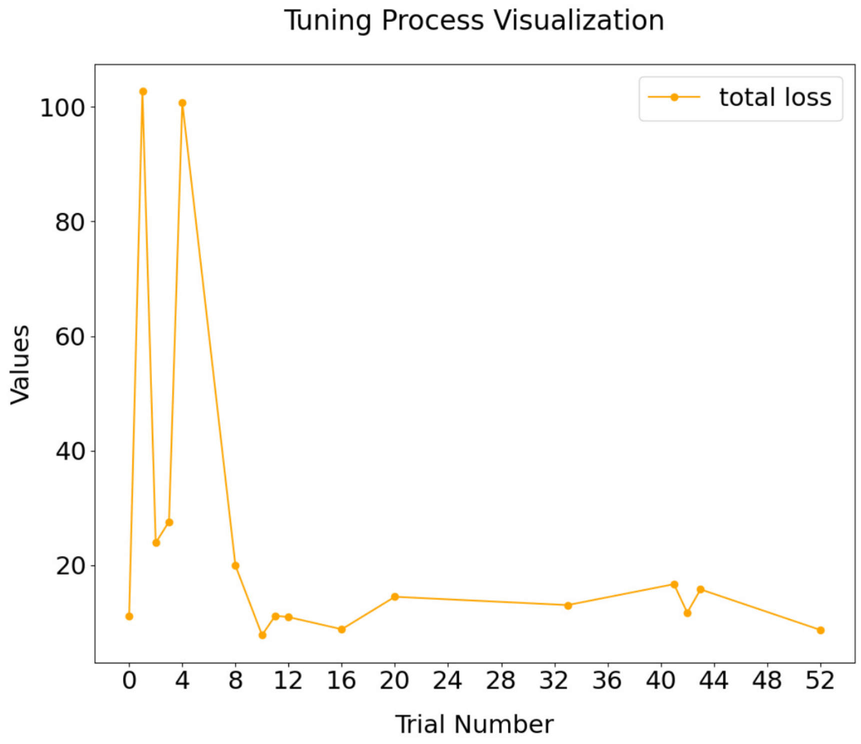

Figure S1: Optimizing process of hyper-parameters in Optuna.

In this study, we apply Optuna (Akiba *et al.*, 2019) to select the best setting of hyper-parameters in our model. Optuna provides a concise method for defining optimization objective and searching space, and supports various advanced optimization algorithms, such as the Tree-structured Parzen Estimator, by which it can efficiently identify the optimal configurations in complex hyper-parameter space. In this study, we define the searching space for three key hyper-parameters of our model: the learning rate was selected from {1e-5, 1e-3, 1e-4, 1e-2}, the noise standard deviation from {0.1, 0.5, 1.0, 1.5, 2.0}, and the number of Transformer layers from {6, 12, 18, 24}. Optuna can automatically determine the optimal combination of hyper-parameters in multiple iterations, and the optimizing process is illustrated in Figure S1. Note that each trial in the horizontal axis of Figure S1 denotes one setting of hyper-parameters. From Figure S1, we can find that our model with the hyper-parameter configuration in the 10th trial derives the best results for this study. The setting of hyper-parameters in the 10th trial is obtained as follows: During the denoising process, noise is added following a normal distribution with a mean of 0 and a standard deviation of 1; During optimization, the learning rate is set to 0.0001; and the Transformer encoder and decoder both consist of 12 stacked layers. Note that other hyper-parameters in our model are determined according to previous research, and the settings are provided in the main manuscript.

3 Supplementary Material for Performance of Training

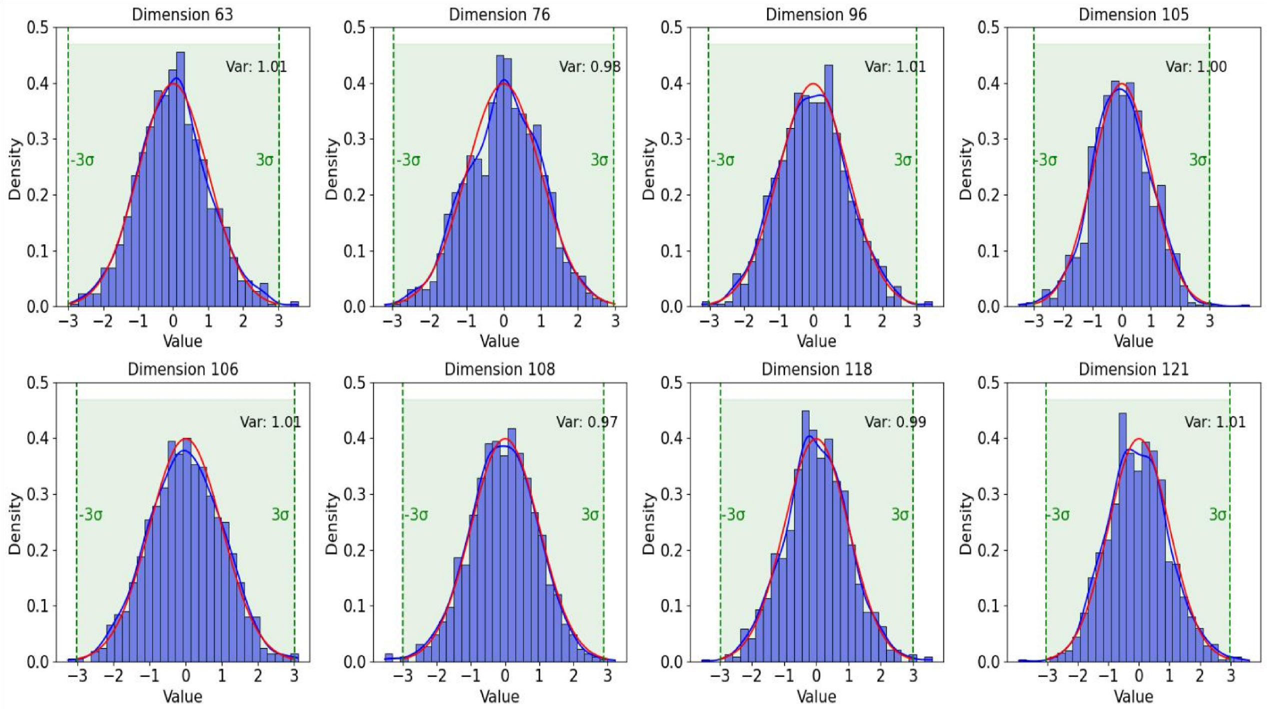

Figure S2: Comparison of latent space distribution and standard normal distribution. The red curve represents the standard normal distribution, while the blue curve illustrates the normal distribution for the corresponding dimensions, which are randomly sampled from eight out of the 128 dimensions. The light green area denotes the 3σ region of the learned distribution.

To verify whether the model has learned the desirable latent representations of the data, we randomly select 8 dimensions from the 128-dimensional latent space and plot the resulting distribution accordingly. For the result in Figure S2, we can find that the variance for dimensions 63, 96, 106 and 121 is just 1.01, showing a high degree of conformity with the standard normal distribution. Moreover, dimension 105 has a variance of 1, which is perfectly aligned with the standard normal distribution, while dimensions 76 and 108 have variances of 0.98 and 0.97, respectively, which although slightly deviating, are still close to the standard normal distribution. Likewise, the data primarily falls within the $\pm 3\sigma$ range, visually indicating that the generated samples align with the expected range of the standard normal distribution. This shows that the sampled data from the latent space conforms to the standard normal distribution without producing too many outliers or extreme values. This results further validates that the model has successfully transformed the data distribution into a standard normal distribution, ensuring that the generated samples are both diverse and conform to the rationality of the normal distribution.

4 Supplementary Material for Ablation Study

We conduct the ablation study to evaluate the contribution of two main components (i.e., the denoising procedure and the property preserving loss) in our model. For performance comparison, three kinds of losses and ratio of high-quality generated AMPs are used as the evaluation metrics. Here, if the average difference between the properties of generated AMP and the target properties (i.e., the input desirable properties) is less than 0.0625, the generated AMP is viewed as the high-quality generated AMPs. For each variant, 1,000 AMPs are generated and the ratio of high-quality generated AMPs is calculated for performance comparison. The results are presented in Table S1, in which "Our model (w/o DN)" denotes

Table S1: Results of Ablation Study.

Methods	PP loss ↓	KL loss ↓	Rec loss ↓	Ratio of high-quality generated AMPs ↑
Our model w/o DN	0.0051	0.0028	4.1240	0.151
Our model w/o PP	0.9956	0.0002	**1.1507**	0.599
Our model	**0.0021**	**0.0002**	1.5956	**0.682**

the variant of our model without the denoising process, and "Our model (w/o PP)" denotes the variant of our model without using the property preserving loss in the optimizing objective.

As can be seen from Table S1, our model clearly derives the best performance by comparing with the two variants, and the ratio of high-quality generated AMPs is about 0.682. For the variant "Our model (w/o DN)", the derived reconstruction loss rises sharply to 4.124, showing that the variant struggles to learn the hidden semantics in validated AMPs. Therefore, it leads to that the ratio of high-quality generated AMPs is only 0.151. The possible reason is that the training samples (i.e., validated AMPs) are limited, and the denoising procedure plays the pivotal role in training an effective AMPs generative model. As for the variant "Our model (w/o PP)", the derived PP loss goes up from 0.0021 (obtained by our model) to 0.9956, showing that the property preserving loss is helpful for deriving AMPs with desirable properties. The derived metric "ratio of high-quality generated AMPs" is also support our observation, which decreases to 0.599.

These findings underscore the importance of the interplay between the denoising procedure and the property preserving loss in optimizing the generative model. Therefore, we can draw the conclusion that two components are both essential for improving the quality of generated AMPs in our model.

5 Supplementary Material for Overall Comparison with Baselines

5.1 Basic Introduction to Baselines

To evaluate the proposed model, we select six representative methods as baselines for performance comparison, which are listed as follows.

- The LSTM method uses the Long Short-Term Memory networks to capture semantics of training samples and generate peptide sequences, which can effectively handle sequential data and capture long-term dependencies.
- The AMP-GAN method introduces additional biological features to guide the generation process, optimizing the generated peptide sequences for both high antimicrobial activity and low toxicity.
- The PepGAN method incorporates biological activity data during the generation process, where the generator network is trained to produce new peptide sequences, and the discriminator network evaluates the authenticity of these sequences, thereby generating effective antimicrobial peptides.
- The WAE method combines the Wasserstein Autoencoder with Particle Swarm Optimization forward search algorithm, primarily for screening anticancer peptides with desired attributes.
- The AMPEMO method proposes an evolutionary multi-objective optimization approach for searching for various antimicrobial peptides. By optimizing multiple objectives, it can effectively discover

and design antimicrobial peptides that meet specific requirements, demonstrating the potential and advantages of multi-objective optimization in antimicrobial peptide discovery.

- The MoFormer method considers conditional generation to produce AMPs with specific hemolysis and minimum inhibitory concentration.

5.2 Detailed Experimental Analysis

Table S2: Performance Comparison among Different Generation Models.

**Methods**	**HEMO <0.5 ↑**	**TOXI <0.5 ↑**
LSTM	0.831	0.630
AMP-GAN	0.393	0.914
Pep-GAN	**0.945**	0.945
WAE	0.338	0.881
AMPEMO	0.443	0.842
MoFormer	0.881	0.936
Our model	0.941	**0.976**

In this study, we evaluate AMPs generation models according to hemolysis and toxicity, which are two critical factors in the development of antimicrobial peptide agents. Specifically, the proportion of sequences generated by our model with $hemolysis < 0.5$ is 0.941, which is the second highest (the difference with the best is merely 0.004) among all models, indicating its effectiveness in minimizing hemolysis risk. Moreover, the proportion of sequences with $toxicity < 0.5$ is 0.976, which is the highest among all models, demonstrating its success in generating AMPs with low-toxicity. To achieve low hemolytic activity and low toxicity, the generated AMPs by our model contain fewer Alanine, Leucine and Proline, which reduces their interaction with the negative charge of the red blood cell membrane, thereby decreasing hemolytic activity. Additionally, the proportion of aromatic residues is mostly around 0.2 (as shown in Figure S5 and the detailed analysis is provided in the following subsection), weakening membrane insertion capability and thus reducing toxicity. Overall, our model excels in maintaining low hemolysis and low toxicity, making it highly promising for clinical applications by providing safe and effective antimicrobial peptides.

6 Supplementary Material for Comparison between Training Samples and Generated AMPs

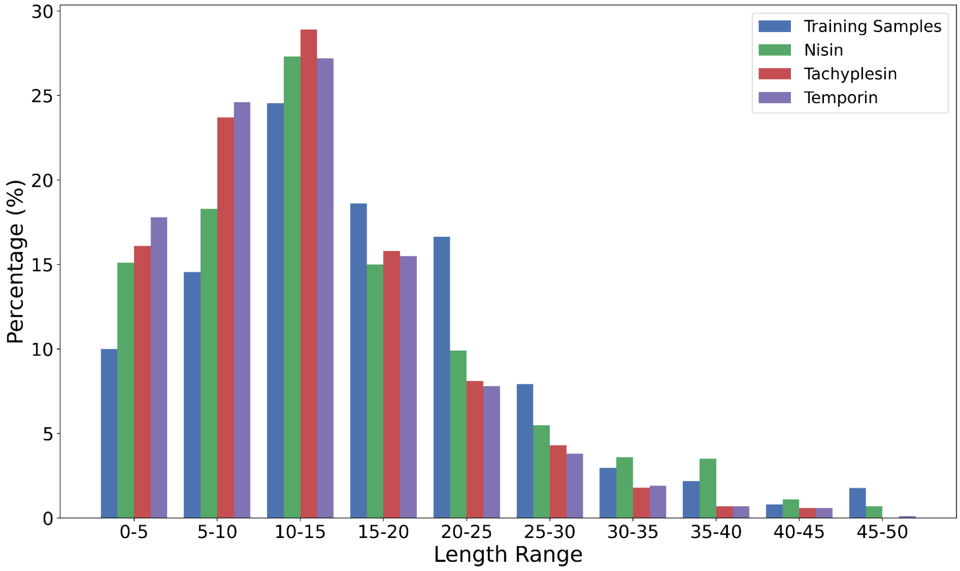

Figure S3: Comparison of distribution of AMPs' lengths between generated and training samples. Three sets of generated AMPs are used, each of which is obtained based on the attributes of Nisin, Tachyplesin, and Temporin, respectively.

First, we analyze the length distribution of generated AMPs. As can be seen from Figure S3, the three length distributions of generated AMPs by our model and the training samples are basically similar, which are mainly concentrated in the range of (0, 20]. This observation is consistent with the typical length range of most effective antimicrobial peptides (Gagat *et al.*, 2024). In addition, generated samples also contain some sequences with more than 20 amino acids. This is due to the different sources of the training set, which includes artificially designed or predicted peptide sequences, resulting in some deviations from the length distribution of AMPs. Generally, the results demonstrate that our model can learn the hidden semantic information contained in the training set from the view of AMPs' lengths.

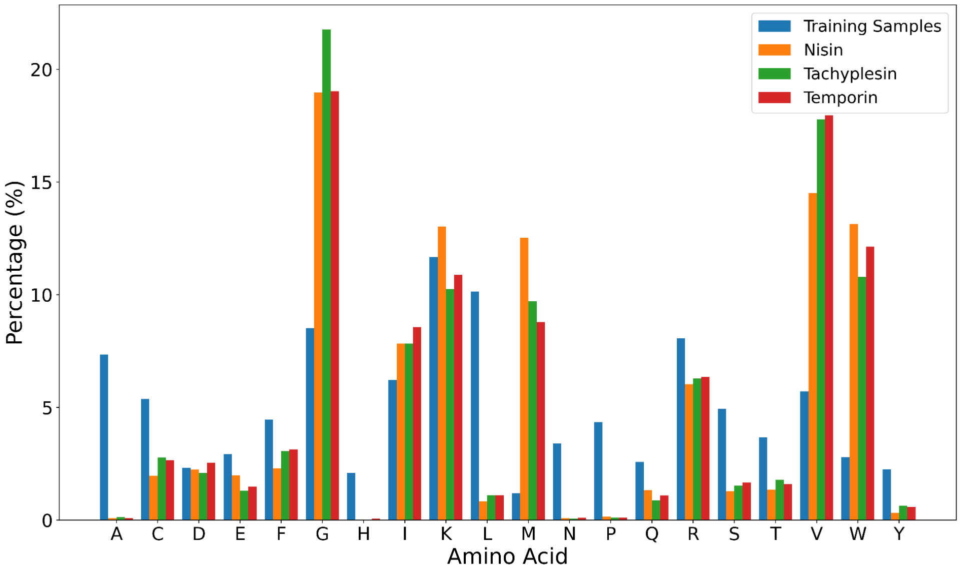

Figure S4: Comparison of amino acid distribution between generated and training samples. Three sets of generated AMPs are used, each of which is obtained based on the attributes of Nisin, Tachyplesin, and Temporin, respectively.

Secondly, we analyze the amino acid compositions between training samples and generated AMPs. From Figure S4, it can be observed that the distribution of amino acids D, E, F, I, K, Q, and R in the training set and generated AMPs by our model is relatively similar, indicating that our model effectively captures the importance of these amino acids in antimicrobial peptides. These amino acids are closely related to the functional characteristics of AMPs. For instance, K and R are positively charged amino acids that increase antibacterial activity by interacting with bacterial cell membranes (Sunar *et al.*, 2024). Similarly, hydrophobic amino acids such as F and I facilitate peptide-membrane interactions (Saint Jean *et al.*, 2018).

Moreover, results in Figure S4 also show that generated AMPs exhibit different patterns for some specific categories of amino acids, such as G, M, V, and W. We argue that this difference will increase the potential of our model to generate novel AMPs. For instance, G, being the smallest amino acid, provides greater structural flexibility to AMPs, which can act as a helix breaker (Sani *et al.*, 2017). M and V, as hydrophobic amino acids, can improve the insertion of AMPs into bacterial membranes, and the study has shown that using these two acids to replace cysteine can maintain the antibacterial activity of AMPs (Nayab *et al.*, 2022). Notably, W has an aromatic structure and can effectively interact with the lipid bilayer, helping peptides to better anchor on the bacterial membrane and increase its ability to penetrate the cell membrane. In addition, the combined use of W and cationic amino acids such as R can form a cation-π interaction, further enhancing the stability and structural integrity of the peptide. (Straus, 2024).

7 Supplementary Material for Comparison of Conditional and Unconditional Generation

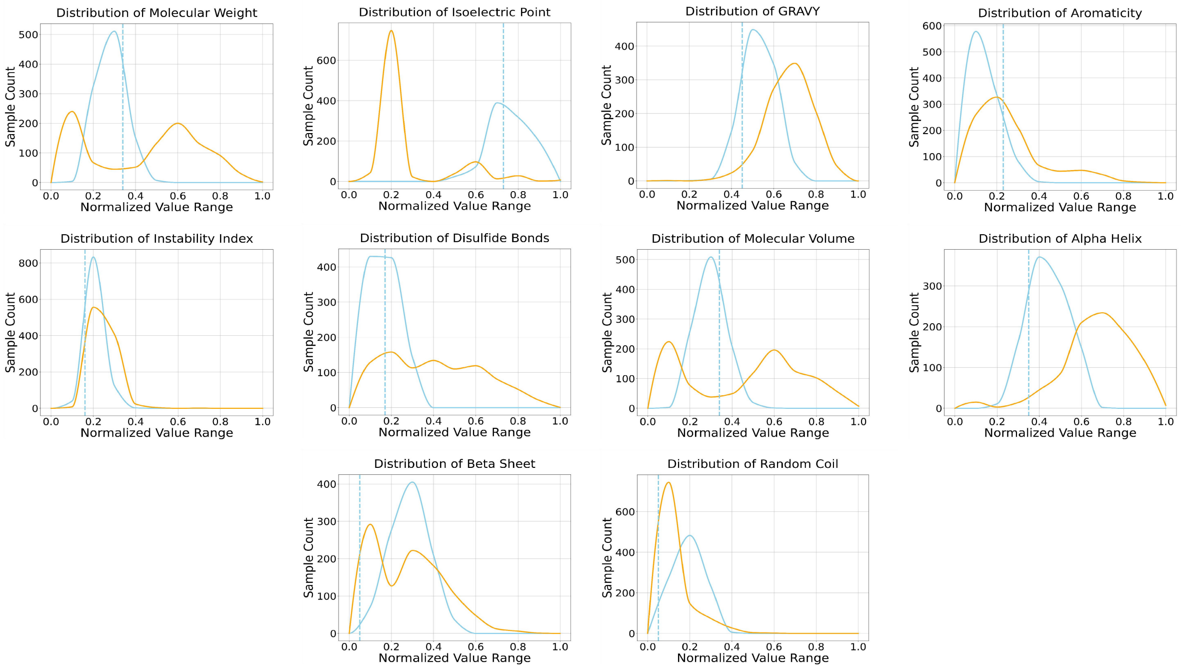

Figure S5: Results of comparison between conditional generation and unconditional generation. The analyzed parameters include molecular weight, isoelectric point, hydrophobicity, aromatic amino acid ratio, instability index, disulfide bonds, molecular volume, and secondary structure of the sequence (α-helix, β-sheet, and random coil). The blue curve denotes the results of conditional generation, while the orange curve denotes the results of unconditional generation. The horizontal axis corresponds to the normalized values of the respective attributes, and the vertical axis indicates the number of instances falling in each range. The dashed vertical line represents the target value for conditional generation. This figure provides a visual comparison of the number of AMPs in each interval range under the two generation modes.

To validate whether the generated AMPs by model exhibit required properties (i.e., the input desirable or expected properties), we compare the distribution of ten physicochemical properties of AMPs obtained by conditional generation and unconditional generation. Specifically, the conditional model shows more concentrated distributions for molecular weight, disulfide bonds, and molecular volume, with values near the target. In contrast, the unconditional model's peak distribution is lower and more dispersed. For the instability index and aromaticity, the distributions of both models are similar, but the conditional model has a significantly higher peak. Additionally, properties such as isoelectric point, and α-helix content are more concentrated around the target values in the conditional model, whereas the unconditional model's peak falls in a different range, indicating the conditional model's better ability to capture desired property values. However, for β-sheet and random-coil in protein secondary structure, the distributions are less concentrated near the target, suggesting that the connection between these properties and sequence information was not fully learned during training. In the future, it is necessary to consider incorporating structural information into the model.

8 Supplementary Material for Conditional Generation Results of Different Conditions

Table S3: Distribution of AMPs under Three Different Conditions.

Target AMP	Molecular Weight	Isoelectric Point	GRAVY	Aromaticity	Instability Index	Disulfide Bonds	Molecular Volume	Alpha Helix	Beta Sheet	Random Coil
Nisin	0.62	0.19	0.57	0.05	0.17	0.03	0.65	0.41	0.14	0.35
Tachyplesin	0.34	0.73	0.45	0.23	0.16	0.17	0.34	0.35	0.05	0.00
Temporin	0.19	0.80	0.48	0.08	0.11	0.03	0.20	0.41	0.25	0.25

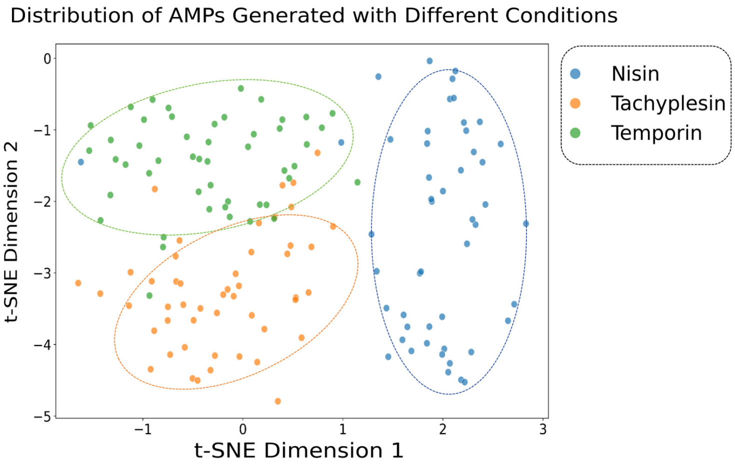

Figure S6: t-SNE dimensionality reduction distribution of ten physicochemical properties of AMPs.

9 Supplementary Material for Discovery of New Antimicrobial Peptides

Table S4: Top 10 Generated AMPs Preserving Most Similar Properties to That of Tachyplesin.

Sequence ID	Sequence	Molecular Weight	Isoelectric Point	GRAVY	Aromaticity	Instability Index	Disulfide Bonds	Molecular Volume	Alpha Helix	Beta Sheet	Random Coil
**Tachyplesin**	**KWCFRVCYRGICYRRCR**	**0.34**	**0.73**	**0.45**	**0.23**	**0.16**	**0.17**	**0.34**	**0.35**	**0.05**	**0.00**
Seq1	VKGIWGMMCRLGR	0.21	0.85	0.55	0.07	0.12	0.11	0.23	0.31	0.23	0.23
Seq2	FGVKFGVRGM	0.15	0.87	0.58	0.20	0.13	0.00	0.16	0.40	0.30	0.10
Seq3	VVGKVIWGMMMRGI	0.23	0.87	0.65	0.07	0.08	0.11	0.24	0.42	0.21	0.21
Seq4	KWGVRVIWKGGMM	0.23	0.89	0.52	0.15	0.14	0.22	0.23	0.38	0.23	0.15
Seq5	VVKKVGGKIGMWM	0.21	0.78	0.58	0.07	0.15	0.11	0.22	0.38	0.23	0.15
Seq6	IKPVMGIGMVMGGKK	0.23	0.78	0.58	0.00	0.15	0.00	0.24	0.27	0.33	0.20
Seq7	VVGGRKIIKMVGWGM	0.24	0.89	0.59	0.06	0.17	0.11	0.25	0.40	0.26	0.13
Seq8	SIIGWGFGMKVRRD	0.24	0.85	0.50	0.14	0.21	0.11	0.24	0.35	0.28	0.07
Seq9	VVGKVFGKKKMMWG	0.23	0.80	0.52	0.14	0.14	0.11	0.25	0.35	0.21	0.14
Seq10	NVKIMWKIGGMGM	0.21	0.74	0.56	0.07	0.14	0.11	0.22	0.31	0.30	0.23

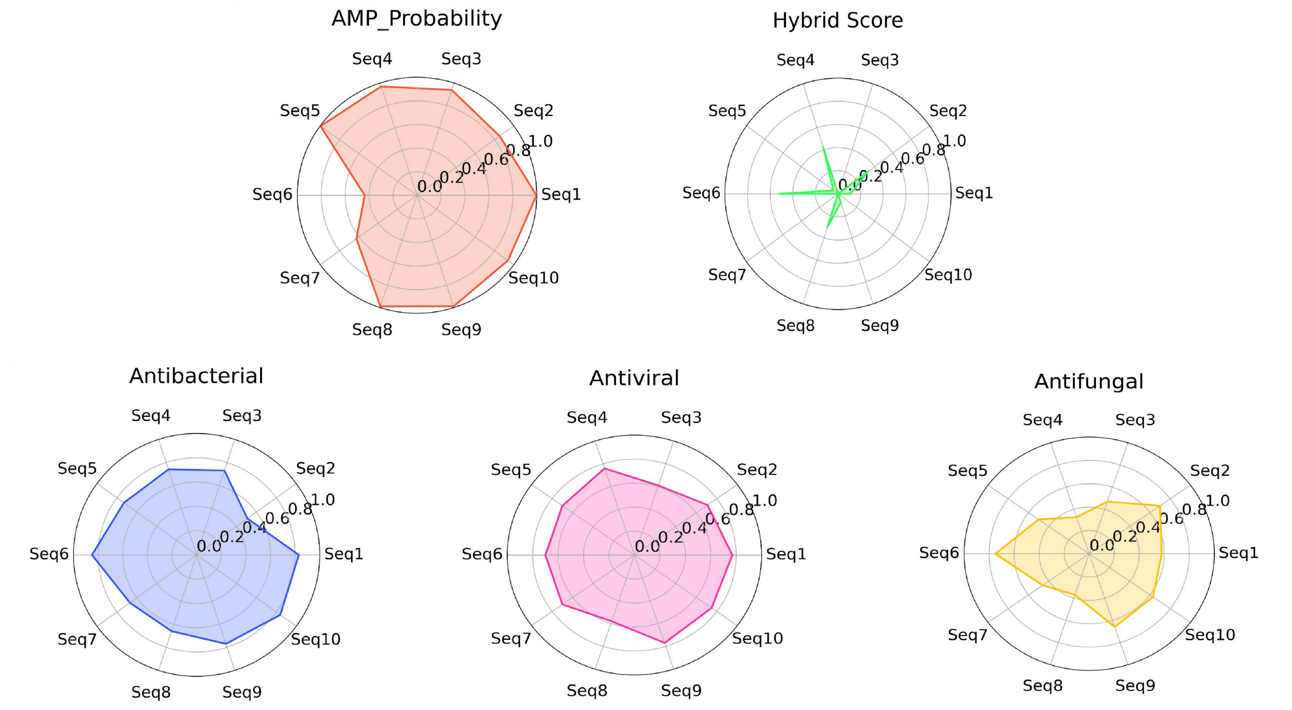

Figure S7: Biochemical properties analysis of ten AMPs. The five radar charts represent AMP probability, toxicity score, antibacterial probability, antiviral probability, and antifungal probability, respectively.

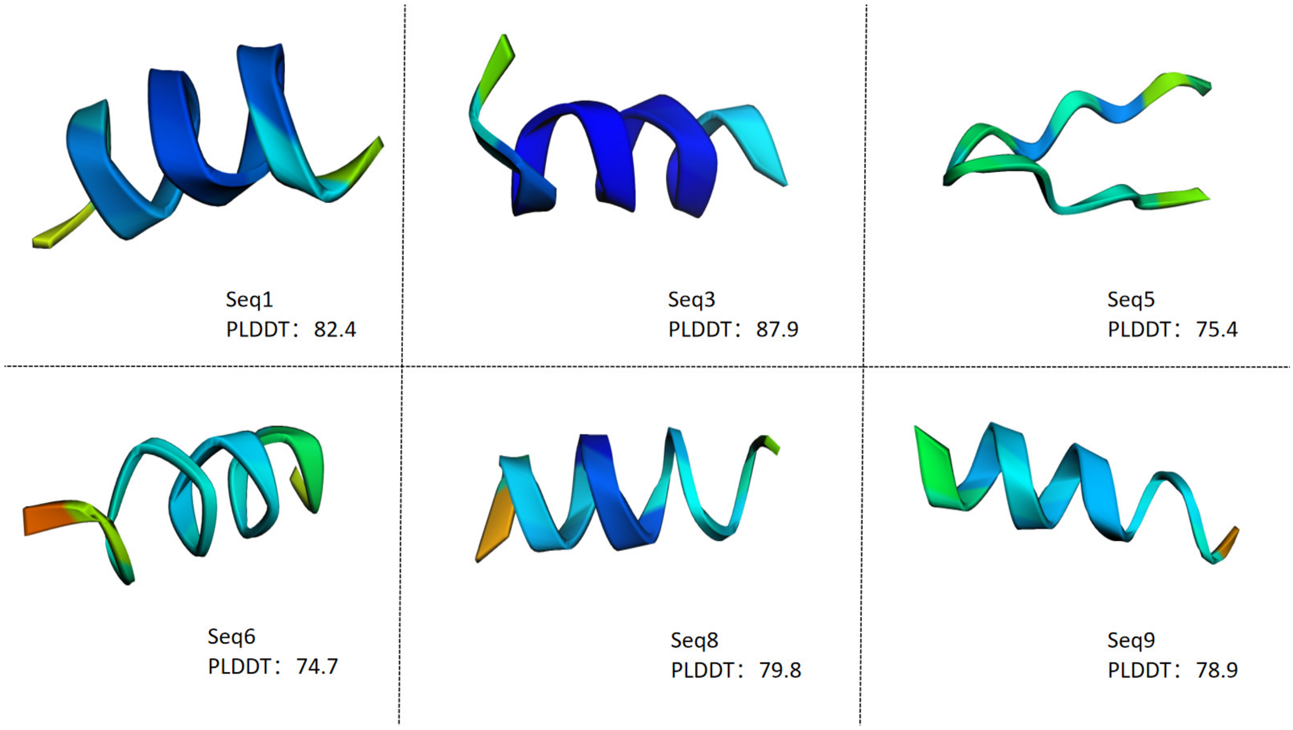

Figure S8: Predicted 3D structures of six AMPs.

To comprehensively evaluate the proposed model's ability to generate novel AMPs, we analyze the biochemical properties of the generated AMPs. From the radar chart analysis in Figure S7, Seq1 and Seq5 demonstrate superior performance across multiple metrics. More specifically, these two AMPs exhibit high AMP probability, antibacterial activity, and antifungal activity, along with low toxicity, making them ideal candidates for further research. Seq2 exhibits the lowest toxicity and relatively high AMP probability, but it is slightly less competitive in other activity metrics. Seq6 shows strong antibacterial and antifungal activity but also demonstrates high toxicity, requiring careful evaluation of its safety. Based

on the predicted 3D structures of several AMPs which are shown in Figure S8, these generated AMPs contain more α-helical conformations. It is known that this structure is critical for AMP functionality, as it facilitates membrane insertion and disrupts membrane integrity, thereby exerting antibacterial, antiviral, or antifungal effects.

References

Akiba, T., Sano, S., Yanase, T., Ohta, T., and Koyama, M. (2019). Optuna: A next-generation hyperparameter optimization framework. In *Proceedings of the 25th ACM SIGKDD international conference on knowledge discovery & data mining*, pages 2623–2631.

Chen, Y., Li, T., Li, J., Cheng, S., Wang, J., Verma, C., Zhao, Y., and Wu, C. (2017). Stabilization of peptides against proteolysis through disulfide-bridged conjugation with synthetic aromatics. *Organic & Biomolecular Chemistry*, **15**(8), 1921–1929.

Gagat, P., Ostrówka, M., Duda-Madej, A., and Mackiewicz, P. (2024). Enhancing antimicrobial peptide activity through modifications of charge, hydrophobicity, and structure. *International Journal of Molecular Sciences*, **25**(19), 10821.

Hitchner, M. A., Santiago-Ortiz, L. E., Necelis, M. R., Shirley, D. J., Palmer, T. J., Tarnawsky, K. E., Vaden, T. D., and Caputo, G. A. (2019). Activity and characterization of a ph-sensitive antimicrobial peptide. *Biochimica et Biophysica Acta (BBA)-Biomembranes*, **1861**(10), 182984.

Jiang, Y., Chen, Y., Song, Z., Tan, Z., and Cheng, J. (2021). Recent advances in design of antimicrobial peptides and polypeptides toward clinical translation. *Advanced Drug Delivery Reviews*, **170**, 261–280.

Nayab, S., Aslam, M. A., Rahman, S. u., Sindhu, Z. u. D., Sajid, S., Zafar, N., Razaq, M., Kanwar, R., and Amanullah (2022). A review of antimicrobial peptides: its function, mode of action and therapeutic potential. *International Journal of Peptide Research and Therapeutics*, **28**(1), 46.

Rydberg, H. A., Carlsson, N., and Nordén, B. (2012). Membrane interaction and secondary structure of de novo designed arginine-and tryptophan peptides with dual function. *Biochemical and biophysical research communications*, **427**(2), 261–265.

Saint Jean, K. D., Henderson, K. D., Chrom, C. L., Abiuso, L. E., Renn, L. M., and Caputo, G. A. (2018). Effects of hydrophobic amino acid substitutions on antimicrobial peptide behavior. *Probiotics and antimicrobial proteins*, **10**, 408–419.

Sani, M.-A., Saenger, C., Juretic, D., and Separovic, F. (2017). Glycine substitution reduces antimicrobial activity and helical stretch of dipgla-h in lipid micelles. *The Journal of Physical Chemistry B*, **121**(18), 4817–4822.

Straus, S. K. (2024). Tryptophan-and arginine-rich antimicrobial peptides: anti-infectives with great potential. *Biochimica et Biophysica Acta (BBA)-Biomembranes*, **1866**(3), 184260.

Sunar, S. Z., Acar, T., and Sahin, F. (2024). Chemically peptide synthesis and role of arginine and lysine in the antimicrobial and antiviral activity of synthetic peptides: a comprehensive review. *Peptide Science*, **116**(5), e24368.

Yang, C.-H., Chen, Y.-L., Cheung, T.-H., and Chuang, L.-Y. (2024). Multi-objective optimization accelerates the de novo design of antimicrobial peptide for staphylococcus aureus. *International Journal of Molecular Sciences*, **25**(24), 13688.
